# Evaluation of the extent of damage to the esophageal wall caused by press-through package ingestion

**DOI:** 10.7717/peerj.6763

**Published:** 2019-04-18

**Authors:** Takuya Tamura, Hajime Okamoto, Toyoaki Suzuki, Yoichi Nakanishi, Daisuke Sugiyama

**Affiliations:** 1 Department of Research and Development of Next Generation Medicine, Faculty of Medical Sciences, Kyushu University, Fukuoka, Japan; 2 Center for Clinical and Translational Research, Kyushu University Hospital, Fukuoka, Japan; 3 Research & Development Center, Fujimori Kogyo CO., LTD., Yokohama, Japan; 4 Department of Clinical Study, Center for Advanced Medical Innovation, Kyushu University, Fukuoka, Japan

**Keywords:** Push through package, Blister pack, Accidental ingestion, Emergency endoscope, Esophageal injury, Patient safety, Packaging design, Bio tribology

## Abstract

Press-through package (PTP) is the most common accidentally ingested foreign body in Japan. Accidental ingestion of PTP can result in esophageal damage. An approach for evaluating the risk of esophageal injury has not been established. Therefore, we used porcine esophageal tissue and silicone sheets to establish a method for assessing the risk of esophageal damage on accidental PTP ingestion. We pathologically evaluated porcine lower esophageal tissue using a scratch tester. Using porcine esophageal tissue, scratch tests were performed with 4 test objects and pathological damage was compared. It was assumed that each object was accidentally ingested. The objects were polyvinylidene chloride (PVDC)-coated polyvinyl chloride (PVC) PTP, soft PThPa, round PTP, and a disposable scalpel. The porcine esophagus was replaced with a silicon sheet, and an automatic friction machine was used for quantitative evaluation. The silicon sheet was scratched using HHS 2000 with 750-g load at 50 mm/min. We investigated the frictional force exerted on the surface for each of the objects. The degree of damage (depth) was the highest for the disposable scalpel, followed by PVDC-coated PVC PTP, while the degree of damage (depth) was the lowest for soft PThPa and round PTP. The mean frictional forces on the silicon sheet were 524.0 gf with PVDC-coated PTP, 323.5 gf with soft PThPa, 288.7 gf with round PTP, and 922.7 gf with the disposable scalpel. We developed approaches to qualitatively and quantitatively evaluate the risk of esophageal damage after accidental PTP ingestion. Our findings indicate that the risk of gastrointestinal damage after accidental PTP ingestion is low with soft PTP and round PTP.

## Introduction

Foreign body ingestion is a common emergency, and in infants younger than 3 years, it has been reported that the risk of accidental ingestion is high (Altkorn et al., 2008; Gregori et al., 2008). Accidental ingestion often occurs even in adults (Birk et al., 2016; Sahn, Mamula & Ford, 2014). In about 80%–90% of cases of accidental ingestion, there is no issue as the foreign body passes spontaneously through the gastrointestinal tract; however, in 10%–20% of cases, removal with endoscopy is necessary. Less than 1% of cases require surgery for extraction of ingested foreign bodies and treatment of complications (Birk et al., 2016; Sahn, Mamula & Ford, 2014; Dray & Cattan, 2013; Pfau, 2014; Sugawa et al., 2014; Telford, 2005). In the literature, among elderly people (>65 years old), most ingested foreign bodies are located in the esophagus (ASGE Standards of Practice Committee et al., 2011). Additionally, in all age groups, the incidence of esophageal foreign body presence has been reported to be as high as 83.3% among individuals who have accidentally ingested a tablet (ASGE Standards of Practice Committee et al., 2011). Recent studies have indicated that press-through package (PTP) represents 29%–38% of accidentally ingested esophageal foreign bodies in Japan (Uehara et al., 2010; Kasugai et al., 2007). Among gastrointestinal tract injuries caused by accidental ingestion of PTP, esophageal injuries are the most common (Kim et al., 2016; Sudo et al., 2003; Norstein et al., 1995). PTP in the esophagus could be difficult to find on radiography, therefore, it is recommended to be diagnosed on CT examination or endoscopy (Kanazawa et al., 2015). Many previous reports have mentioned perforation rates as high as 35% after ingestion of sharp objects, and in severe cases, esophageal perforation has been shown to be accompanied with various complications (Birk et al., 2016; Sahn, Mamula & Ford, 2014; Kim et al., 2016; Sudo et al., 2003; Norstein et al., 1995; Kanazawa et al., 2015; Zou et al., 2011; Ambe et al., 2012; Laeeq et al., 2015; Law et al., 2017; Geraci et al., 2016; Burgos, Rabago & Triana, 2016). In 2010, the Ministry of Health, Labor and Welfare issued a notice on accidental swallowing of PTP to the Japan Pharmaceutical Federation, the Japanese Pharmaceutical Association, and the Japan Hospital Pharmacists Association (Ministry of Health, Labour and Welfare of Japan, 2010; Japan Council for Quality Health Care, 2011; Japan Council for Quality Health Care, 2013). In the notification, there were requests to consider measures to round the PTP corners and reduce the burden on the body if PTP was accidentally swallowed. Since then, improvements have been made to the shape and material of PTP. However, an approach to evaluate the risk of esophageal injury after accidental PTP ingestion has not been established. Therefore, we used porcine esophageal tissue, which is similar to human esophageal tissue (Christie, Thomson & Hopwood, 1995), to establish a method for assessing the risk of esophageal damage on accidental PTP ingestion, and we pathologically evaluated porcine lower esophageal tissue using a scratch tester.

## Materials & Methods

The present study involved two types of experiments (qualitative evaluation in manual scratch tests and quantitative evaluation in automatic scratch tests).

### Objects

**Figure 1:**
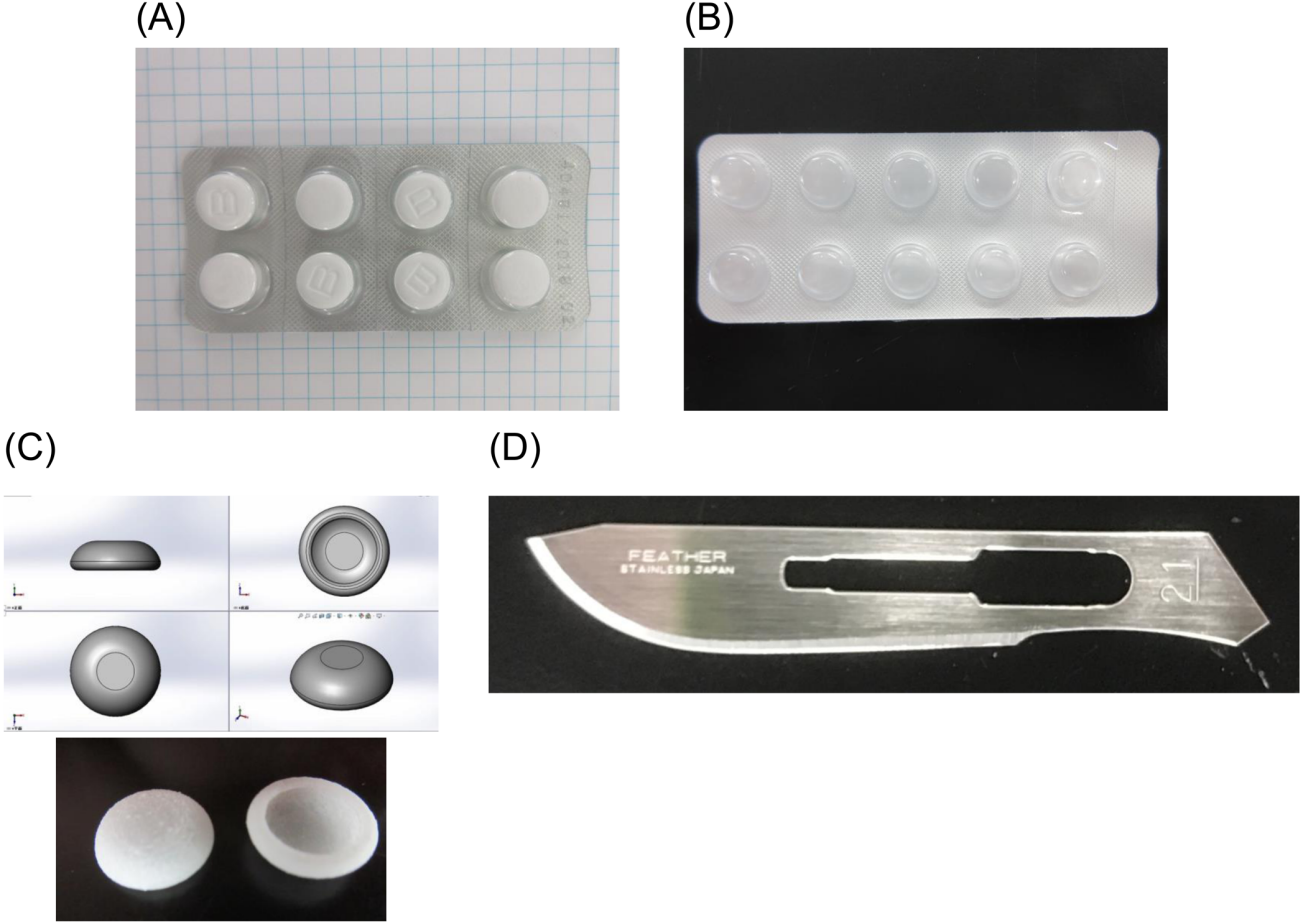
Objects. (A) PVDC-coated PVC PTP, (B) soft PThPa, (C) round PTP, (D) disposable scalpel.

The following four objects were included: (1) polyvinylidene chloride (PVDC)-coated polyvinyl chloride (PVC) PTP (popular PTP), (2) soft PThPa (soft material PTP by Fujimori Kogyo Co., Ltd., Japan), (3) round PTP (3D-printed PTP made using PVC material), and (4) a disposable scalpel (Feather No. 21; thickness 0. 3 × blade length 26 mm) (Fig. 1). Using a fixture, scratch tests were conducted under a normal load of 750 g.

### Manual scratch test and pathology

Isolated porcine lower thoracic esophageal specimens, which were obtained from Yokohama meat market, were cut and opened. Using these porcine esophageal specimens, we conducted manual scratch tests to compare PVDC-coated PVC PTP, soft PThPa, round PTP, and a disposable scalpel, with 750 g as the normal load. The scratch test was performed according to ISO 15184:2012E (paints and varnishes—determination of film hardness by a pencil test) and JIS K 5600-5-4. We modified the Clemen scratch tester by fixing the objects to the tester. This study was conducted at The Institute of Fujimori Kogyo Co., Ltd. All experiments were performed under stable conditions (room temperature, 22 °C–23 °C; room humidity, 45%–50%). This study did not include an animal experiment requiring permission of IRB according to the animal ethics guidelines of Kyushu University.

**Figure 2:**
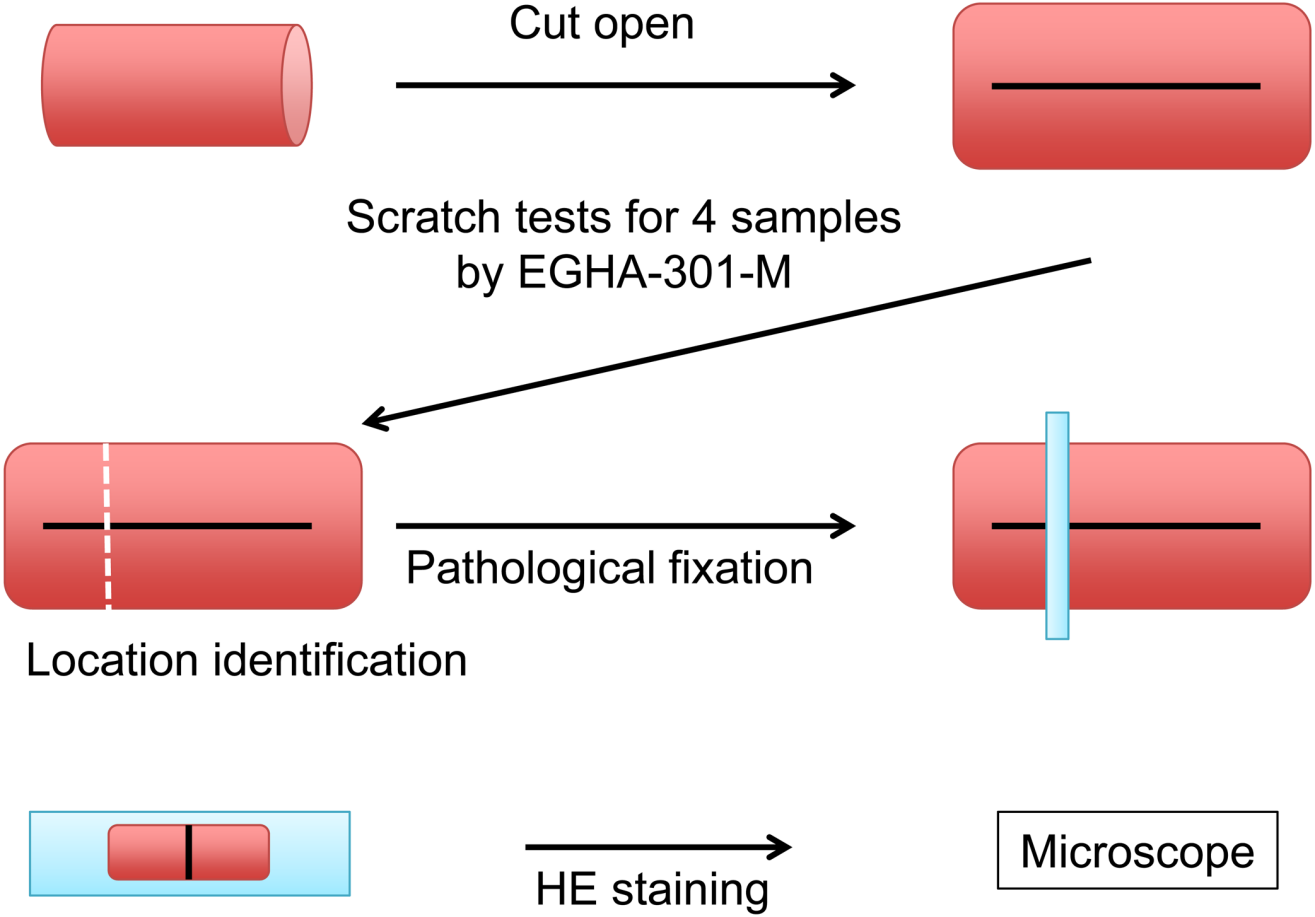
Schema of the scratch test and pathological procedure on the porcine esophagus.

We used a manual Clemen scratch tester (Model No. EGHA-301-M) that conforms to ISO15184:2012E and JIS K 5600-5-4. The fixture of the objects was designed to apply the normal load at an angle of 45 degrees. A corkboard was used to fix the tissue. A fixed base with an acrylic plate embedded in the center was used so that the corkboard would not become a cushion to relieve pressure when performing the scratch test (Fig. 2). Each object was moved from the pharyngeal side to the stomach side at a speed of 50 mm/min, with a weight of 750 g. The tested tissues were fixed in 10% formalin, cut in a direction transverse to the scratches, and embedded in paraffin. The embedded tissues were cut in 3-µm sections. The sections were placed on slides, stained with hematoxylin and eosin (HE), and imaged.

### Automatic scratch test

The relationship between the normal load and dynamic frictional force was investigated using an automatic friction tester (HHS 2000) (Noriyasu et al., 2005). According to previous literature, the mucous membrane of the digestive tract can be substituted with silicon for simulation in education and basic experiments. In this experiment, we aimed to establish an evaluation method that does not use living organisms and to quantitatively compare frictional force using a silicon sheet (Dresselhuis et al., 2008; Ranc et al., 2006; Ujiie et al., 2017). In order to compare the frictional force with the objects, we quantified the frictional force. The sampling rate was 100/s. The conditions were as follows: scratch over a distance of 50 mm at a speed of 0.8 mm/min with a fixed normal load of 750 g.

### Outcomes and data analysis

The esophagus is divided into the lumen, mucosal epithelium, mucosal lamina propria, muscularis mucosa, submucosa, muscle layer (inner annular layer and outer longitudinal layer), and adventitia (Kuo & Urma, 2006). HE staining was performed on the tested area, and the histological deep layer was evaluated. Each assessment was performed thrice. In the 750-g fixed load tests, the mean frictional force was determined from data of 40 mm of the specimen, excluding the unstable first 10 mm. The mean frictional force of each object was assessed with Tukey’s HSD test using JMP 13.0 (SAS Institute Inc., Cary, NC, USA). From this analysis, the *P* value when assuming that there is no difference between individual groups was described, and the comparison result was shown with *p* < 0.05 as the rejection criterion.

## Results

**Figure 3:**
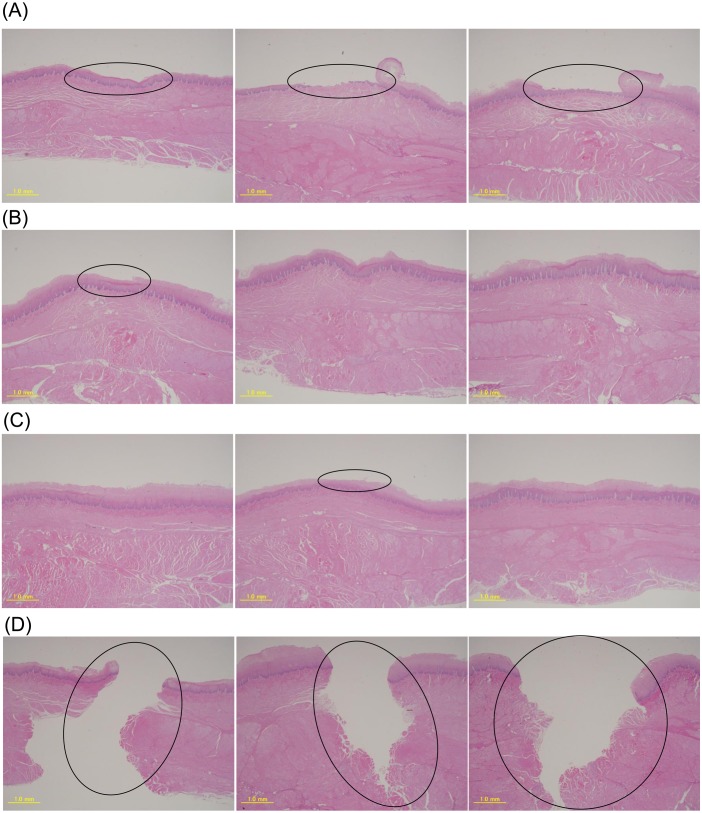
Pathological results for the 4 objects by EGHA-301-M. (A) PVDC-coated PVC PTP, (B) soft PThPa, (C) round PTP, (D) disposable scalpel.

**Figure 4:**
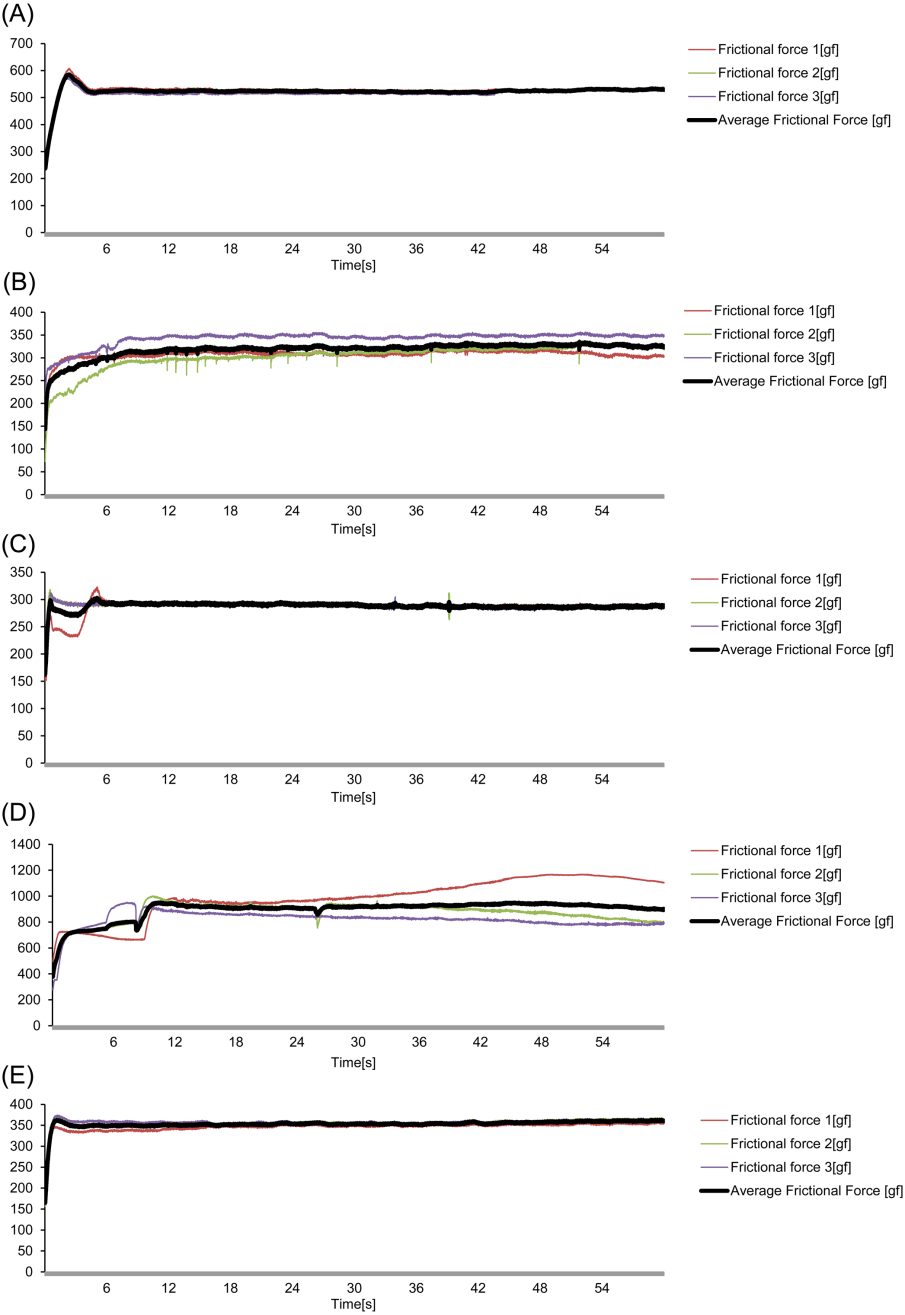
The frictional force in the 750-g fixed load scratch test for the 4 objects and the 0.6-mm-diameter sapphire needle using HHS 2000. (A) PVDC-coated PVC PTP, (B) soft PThPa, (C) round PTP, (D) disposable scalpel, (E) sapphire needle.

**Figure 5:**
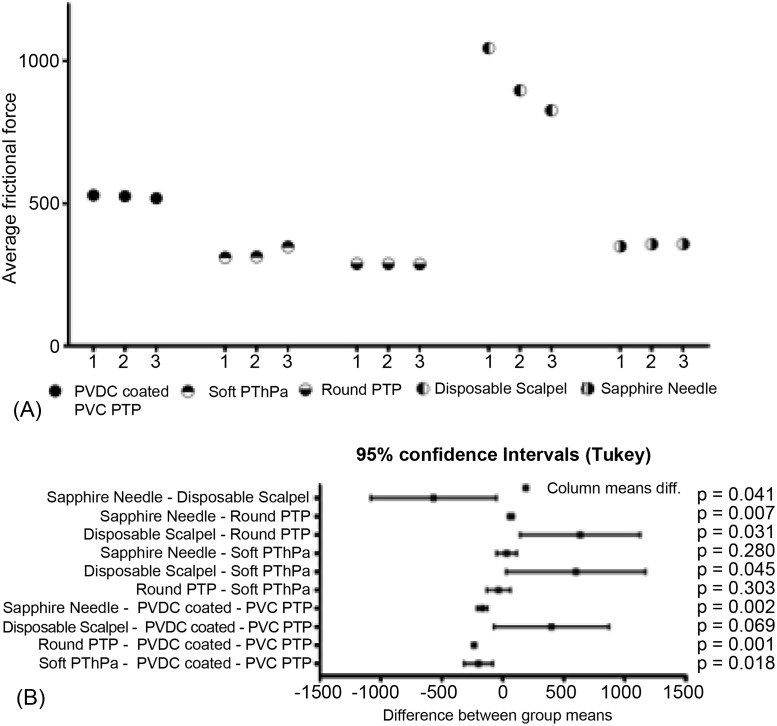
Comparison of the mean frictional force generated by the four objects and the sapphire needle. (A) Three data sets as average frictional force obtained from each of the five groups. (B) Comparison of data sets by five groups.

Pathological evaluation and comparison by quantitative evaluation were possible for four objects respectively. Moreover, the results are consistent with qualitative evaluation and quantitative evaluation. On pathological evaluation, it was found that the mucosal epithelium was damaged in all three assessments of PVDC-coated PVC PTP, and in one of these assessments, damage to the basement membrane was noted. With regard to soft PThPa and round PTP, of the three assessments for each object, 1 showed damage to the stratum corneum of the mucosal epithelium. With regard to the disposable scalpel, all three assessments showed penetration of the adventitia (Fig. 3, Table 1). The results of the 750-g load scratch test using the four objects and a sapphire needle as the quantitative evaluation were shown in Fig. 4. The mean, minimum, and maximum values obtained from 40 mm of tissue, excluding the unstable first 10 mm are summarized in Table 2. The average frictional force of the four test object groups and the sapphire needle were compared (Fig. 5). From Tukey’s HSD test, disposable scalpel >PVDC-coated PVC PTP >round PTP or soft PThPa, and sapphire needle >round PTP. There was no significant difference between soft PThPa and round PTP. In the scratch test using a scalpel, as the silicon sheet was penetrated, the friction with the stainless steel jig used for fixation was measured, and this was considered as the reference value. These pathological and quantitative evaluation were consistent that the higher damage risk was ingestion of disposable scalpel >PVDC-coated PVC PTP >soft PThPa and round PTP in this order.

## Discussion

When esophageal damage occurs beyond the basal layer of the mucosa, clinical gastrointestinal bleeding, erosion, and inflammatory cell infiltration can occur; thus, damage beyond the mucosal epithelium may be considered a problematic digestive tract injury (Kuo & Urma, 2006). From an anatomical point of view, it is possible to explain clinically that gastrointestinal bleeding occurs when the lesion crosses the basement membrane. The lower thoracic esophagus is nourished by branches of the proper esophageal artery and the intercostal arteries (Kuo & Urma, 2006; Goyal & Hiroshi, 2006; Hiroshi & Goyal, 2006). The proper esophageal artery branch that reaches the esophageal adventitia branches out to a capillary network distributed in the adventitial membrane, branches off, penetrates the external longitudinal muscle layer, and forms a dense arterial network in the inner circular muscle layer (Kuo & Urma, 2006). The artery coming out from this region enters the submucosal layer by penetrating the circular muscle. In the submucosal layer, the arteries are anastomosed to each other, forming an irregular arterial network extending in the longitudinal direction. A large number of small arteries extend from the arterial network, penetrate the lamina muscularis mucosae, enter the proper mucosal layer, and divide into several small branches forming a capillary network. Additionally, venous capillaries collectively form venules and form a vascular network that runs longitudinally at equal intervals within the proper mucosal layer. A vein that exits from the vascular network penetrates the muscularis mucosa plate, forms a venous network in the submucosal layer, merges with the neighboring venous network, passes through the muscular layer, and reaches the adventitia (Kuo & Urma, 2006; Goyal & Hiroshi, 2006; Hiroshi & Goyal, 2006). Thus, the basement membrane is a clinically problematic boundary. Sharp foreign bodies, such as PTP, needles, and disposable scalpels, were classified into the same category clinically, but we confirmed differences in the degree of damage (depth) among these foreign bodies under our experimental conditions (Pfau, 2014; Sugawa et al., 2014; Telford, 2005; ASGE Standards of Practice Committee et al., 2011; Uehara et al., 2010; Kasugai et al., 2007). In our pathological examination using porcine esophageal tissue, the scratch test of each object showed that the degree of damage (depth) was the highest for the disposable scalpel, followed by PVDC-coated PVC PTP, while the degree of damage (depth) was the lowest for soft PThPa and round PTP.

**Table 1:** Pathological results of the 750-g scratch tests for the 4 objects.

Objects	Trial No.	Depth of injury
PVDC-coated PVC PTP	1	stratum corneum
	2	basement membrane
	3	stratum basale
Soft PThPa	1	stratum corneum
	2	intact
	3	intact
Round PTP	1	intact
	2	stratum corneum
	3	intact
Disposable scalpel	1	adventitia
	2	adventitia
	3	adventitia

With the normal load of 750 g, soft PThPa and round PTP showed injury that did not exceed the stratum corneum of the mucosal epithelium. On the other hand, the PVDC-coated PVC PTP showed damage exceeding the basement membrane, while the disposable scalpel showed penetration of the adventitia.

The frictional force that exceeded the basement membrane was 324 gf or more and 524 gf or less according to the pathological result obtained from the 3 objects and the frictional force quantified by the 750-g scratch test using a silicone sheet.

In the comparison between soft PThPa and PVDC-coated PVC PTP, soft PThPa had a shallower damage depth, and in the comparison between PVDC-coated PVC PTP and round PTP, round PTP had a shallower damage depth. Thus, soft material and round shape were considered to reduce the risk of esophageal damage on accidental ingestion.

As PTP made of soft material has some elasticity against pressure, the force at the corner is dispersed on contact and the local frictional force reduces. Therefore, the depth of damage of soft PTP was low. Additionally, for round PTP, the surface area in contact with the esophagus is high, and thus, the local frictional force reduces.

**Table 2:** The mean, maximum, and minimum frictional force values on scratching a silicon sheet at 750 g.

Objects	Frictional force	First time	Second time	Third time	Mean
PVDC-coated PVC PTP	Minimum	521	517	508	517
	Maximum	538	536	540	533
	Mean	529	526	518	524
Soft PThPa	Minimum	298	262	339	307
	Maximum	328	340	356	335
	Mean	310	313	348	323
Round PTP	Minimum	282	263	278	279
	Maximum	297	312	305	296
	Mean	289	289	288	289
Disposable scalpel	Minimum	927	754	770	856
	Maximum	1168	999	911	951
	Mean	1045	897	827	923
Sapphire needle	Minimum	335	348	349	347
	Maximum	358	368	367	363
	Mean	349	357	358	355

By applying the approach used in this study, it is possible to evaluate esophageal damage risk for other objects. If the frictional force of a silicone sheet at 750-g fixed load is 324 gf or less, it can be considered that the risk of damage exceeding the esophageal basement membrane is low.

From the continuous load variation friction testing experiment (Figure S4) as the validation of porcine and silicone sheets, the regression line was *Y* = 0.558X (X was the load and Y was the frictional force, *R*^2^ = 0.994). The estimated frictional force on silicone sheets obtained by substituting *X* = 750 g into the regression line of the sapphire needle was *Y* = 419 gf. In the 750-g load test, the true mean frictional force of the sapphire needle was 355 gf. The coefficient corrected from the 750-g fixed load test to the result of the continuous load variation friction test was 0.848. In the continuous load test, the damage risk for each object can be evaluated by varying the load from 0 g to 800 g and comparing the load when the frictional force reaches 382 gf or 618 gf.

## Limitations

To perform experiments with the human esophagus, obtaining the esophagus in a living state or immediately after death is crucial, but this is difficult and may pose ethical problems. In this study, we used the porcine esophagus instead of the human esophagus as a model for biological experiments. Furthermore, since there are individual differences in the porcine esophagus, we examined the substitution with silicone sheet, which can obtain consistent and reliable data. On the basis of these facts, we confirmed the validity of the substitution; however, our findings may not be applicable to the reactions in the actual human esophagus. Using our method, the risk of damage to the esophagus caused by the ingestion of objects, could be assessed, which has not been studied to date. The pressures identified are not the actual esophageal pressures but the experimental pressures when a fixed load is applied. In addition, this approach is a modified test of ISO 15184:2012E, and the conditions of load setting and speed are different from the conditions when a human accidently ingests a foreign body. The bolus food travel time through the esophagus has been reported to be 5–6 s, with a peristalsis velocity of 3–4 cm/s (Goyal & Hiroshi, 2006). Frictional force is generated on the surface with concentration at the contact area, and it depends on the area where internal esophageal pressure is applied, including reactions such as neural reflex and cough (Kuo & Urma, 2006; Goyal & Hiroshi, 2006; Hiroshi & Goyal, 2006; Vegesna et al., 2013). For example, the spontaneous cough pressure of a healthy adult male is about 180 cm H_2_O, and depending on the condition of an individual, it may exceed 300 cm H_2_O (Lee et al., 2015). The experiments were performed to compare the risk of physical damage among the assessed objects and not to reproduce practical accidental ingestion.

## Conclusions

We developed approaches to qualitatively and quantitatively evaluate the risk of esophageal damage after accidental PTP ingestion. There were no differences in the results of pathological evaluation (qualitative) performed using porcine esophageal tissue and tribological evaluation (quantification) performed using a silicone sheet. The damage risk identified was comparable between the approaches. In both qualitative and quantitative evaluations, the damage risk was lower with soft PTP and round PTP than with PVDC-coated PTP. The risk of esophageal damage associated with accidental PTP ingestion might be reduced by using soft material and designing round PTP.

##  Supplemental Information

10.7717/peerj.6763/supp-1 Figure S1 Schema of the manual scratch test The schema that was manually scratch tests are shown in Figure S1.

10.7717/peerj.6763/supp-2 Figure S2 Schema of the automatic friction test (Tribometer HHS 2000) The schema that was automatic scratch tests are shown in FigureS2.

10.7717/peerj.6763/supp-3 Figure S3 Schema of the scratch test of the 4 objects using HHS 2000 with silicon sheets fixed on the stage The method of fixing the silicon and the test object in the automatic scratch test is shown in FigureS3.

10.7717/peerj.6763/supp-4 Figure S4 The relationship between the normal load and frictional force in the porcine esophageal tissue and the silicone sheet for a 0.6-mm-diameter sapphire needle using HHS 2000 The characteristics of porcine esophageal tissue for continuous load variation friction testing were clarified, and calibration curves were prepared. The same experiments were performed on the silicone sheet. Condition; scratch over a distance of 50 mm at a speed of 0.8 mm/min while changing the normal load from 0 g to 800 g, 42 times for the porcine esophageal tissue (Figure S4-A), 3 times for the silicone sheet (Figure S4-B). The raw data were in Dataset S1 and Dataset S2.
From the results, the regression line of the silicon sheet was *Y* = 0.588*X* − 16.4 (*X* was the load and *Y* was the frictional force, *R*^2^ = 0.998). The regression line of the porcine esophageal tissue was *Y* = 0.543*X* − 10.7 (*X* was the load and Y was the frictional force; *R*^2^ = 0.998). The regression line obtained by correcting the intercept to 0 was *Y* = 0.558*X* (*R*^2^ = 0.994) and *Y* = 0.523*X* (*R*^2^ = 0.996), and the slope ratio was 1.07.

10.7717/peerj.6763/supp-5 Figure S5 The relationship between the normal load and mean coefficient of friction (COF) in the porcine esophageal tissue and silicone sheet for a 0.6-mm-diameter sapphire needle using HHS 2000 The vertical axis is COF and the horizontal axis is plotted as load in FigureS5 from FigureS4 and DatasetS3.

10.7717/peerj.6763/supp-6 Figure S6 Pathological picture1 (PVDC-coated PVC PTP trial No.1) The depth of injury is stratum corneum.

10.7717/peerj.6763/supp-7 Figure S7 Pathological picture 2 (PVDC-coated PVC PTP trial No.2) The depth of injury is basement membrane.

10.7717/peerj.6763/supp-8 Figure S8 Pathological picture 3 (PVDC-coated PVC PTP trial No.3) The depth of injury is stratum basale.

10.7717/peerj.6763/supp-9 Figure S9 Pathological picture 4 (Soft PThPa trial No.1) The depth of injury is stratum corneum.

10.7717/peerj.6763/supp-10 Figure S10 Pathological picture 5 (Soft PThPa trial No.2) The pathological evaluation is intact.

10.7717/peerj.6763/supp-11 Figure S11 Pathological picture 6 (Soft PThPa trial No.3) The pathological evaluation is intact.

10.7717/peerj.6763/supp-12 Figure S12 Pathological picture 7 (Round PTP trial No.1) The pathological evaluation is intact.

10.7717/peerj.6763/supp-13 Figure S13 Pathological picture 8 (Round PTP trial No.2) The depth of injury is stratum corneum.

10.7717/peerj.6763/supp-14 Figure S14 Pathological picture 9 (Round PTP trial No.3) The pathological evaluation is intact.

10.7717/peerj.6763/supp-15 Figure S15 Pathological picture 10 (Disposable scalpel trial No.1) The depth of injury is adventitia.

10.7717/peerj.6763/supp-16 Figure S16 Pathological picture 11 (Disposable scalpel trial No.2) The depth of injury is adventitia.

10.7717/peerj.6763/supp-17 Figure S17 Pathological picture 12 (Disposable scalpel trial No.3) The depth of injury is adventitia.

10.7717/peerj.6763/supp-18 Dataset S1 Automatic scratch test by sapphire to porcine sheet raw data1 Condition; scratch over a distance of 50 mm at a speed of 0.8 mm/min while changing the normal load from 0 g to 800 g, 42 times for the porcine esophageal tissue.

10.7717/peerj.6763/supp-19 Dataset S2 Automatic scratch test by sapphire to silicone sheet raw data2 Condition; scratch over a distance of 50 mm at a speed of 0.8 mm/min while changing the normal load from 0 g to 800 g, 3 times for the silicone sheet.

10.7717/peerj.6763/supp-20 Dataset S3 Raw data for comparison of porcine and silicon sheets (FigureS5)

10.7717/peerj.6763/supp-21 Dataset S4 Automatic scratch test by PVDC-coated PVC on silicone sheet raw data 1

10.7717/peerj.6763/supp-22 Dataset S5 Automatic scratch test by PVDC-coated PVC on silicone sheet raw data 2

10.7717/peerj.6763/supp-23 Dataset S6 Automatic scratch test by PVDC-coated PVC on silicone sheet raw data 3

10.7717/peerj.6763/supp-24 Dataset S7 Automatic scratch test by Soft PThPa on silicone sheet raw data 1

10.7717/peerj.6763/supp-25 Dataset S8 Automatic scratch test by Soft PThPa on silicone sheet raw data 2

10.7717/peerj.6763/supp-26 Dataset S9 Automatic scratch test by Soft PThPa on silicone sheet raw data 3

10.7717/peerj.6763/supp-27 Dataset S10 Automatic scratch test by round PTP on silicone sheet raw data 1

10.7717/peerj.6763/supp-28 Dataset S11 Automatic scratch test by round PTP on silicone sheet raw data 2

10.7717/peerj.6763/supp-29 Dataset S12 Automatic scratch test by round PTP on silicone sheet raw data 3

10.7717/peerj.6763/supp-30 Dataset S13 Automatic scratch test by sapphire on silicone sheet raw data 1

10.7717/peerj.6763/supp-31 Dataset S14 Automatic scratch test by sapphire on silicone sheet raw data 2

10.7717/peerj.6763/supp-32 Dataset S15 Automatic scratch test by sapphire on silicone sheet raw data 3

10.7717/peerj.6763/supp-33 Dataset S16 Automatic scratch test by disposable scalpel on silicone sheet raw data 1

10.7717/peerj.6763/supp-34 Dataset S17 Automatic scratch test by disposable scalpel on silicone sheet raw data 2

10.7717/peerj.6763/supp-35 Dataset S18 Automatic scratch test by disposable scalpel on silicone sheet raw data 3

## Additional Information and Declarations

### Data Availability

The following information was supplied regarding data availability: Raw data can be found in the Supplemental Information.
