# Supplementary figures and images for: Evaluation of the extent of damage to the esophageal wall caused by press-through package ingestion
Source: PeerJ. 2019 Apr 18;7:e6763. doi: 10.7717/peerj.6763 (PMC6475578; doi:10.7717/peerj.6763)

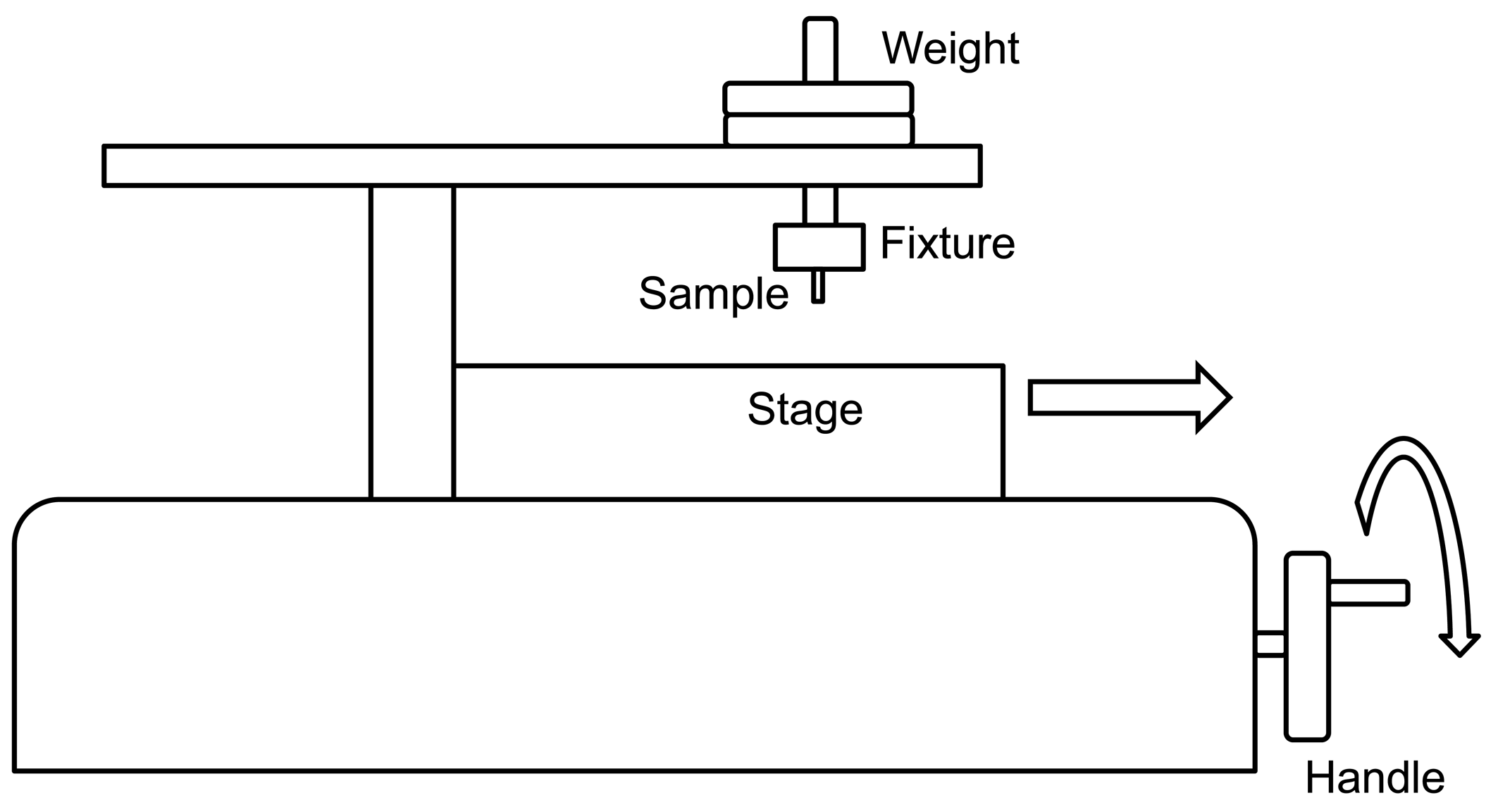

Supplement: Figure S1 — The schema that was manually scratch tests are shown in Figure S1. [file peerj-07-6763-s001.png]

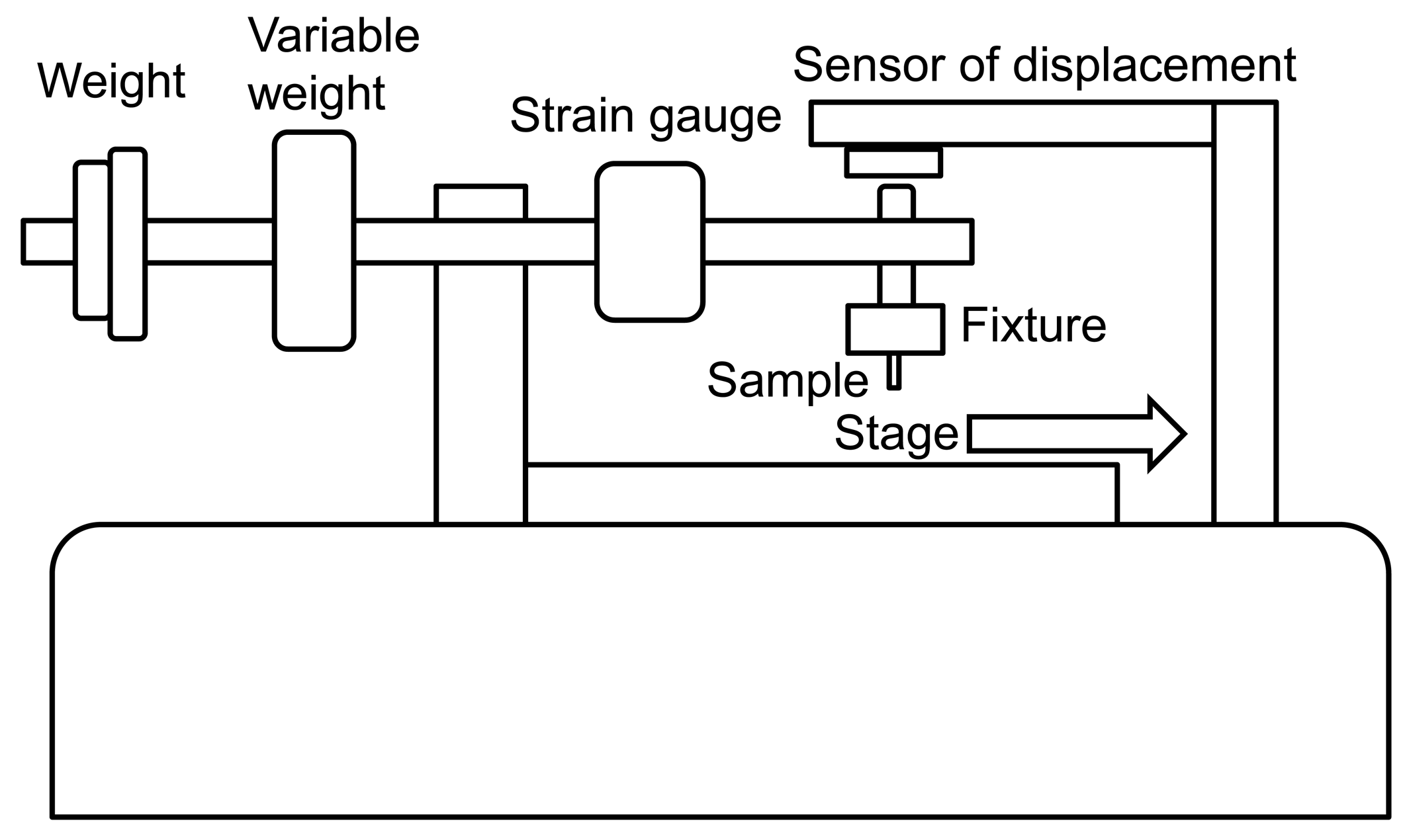

Supplement: Figure S2 — The schema that was automatic scratch tests are shown in FigureS2. [file peerj-07-6763-s002.png]

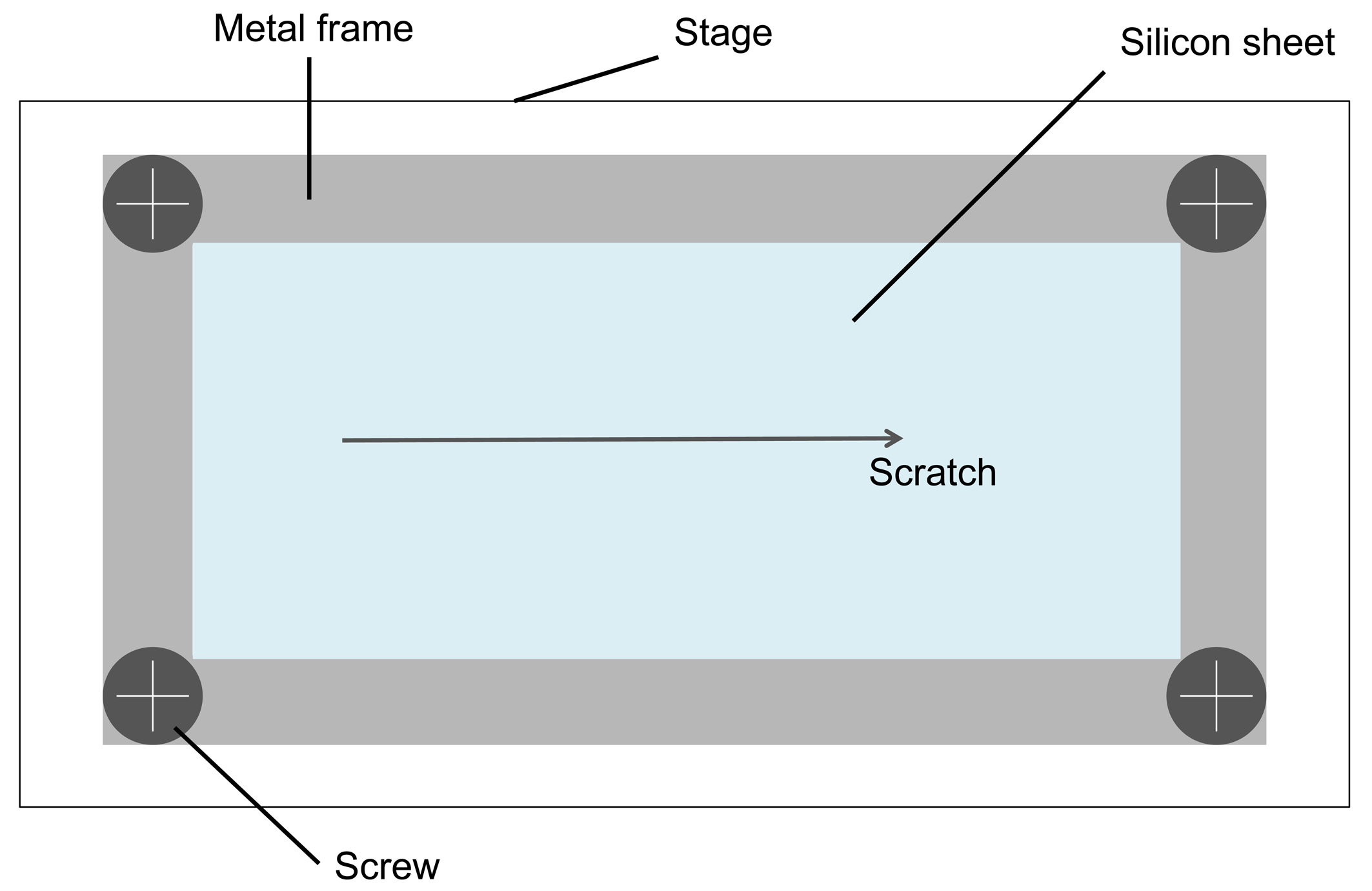

Supplement: Figure S3 — The method of fixing the silicon and the test object in the automatic scratch test is shown in FigureS3. [file peerj-07-6763-s003.png]

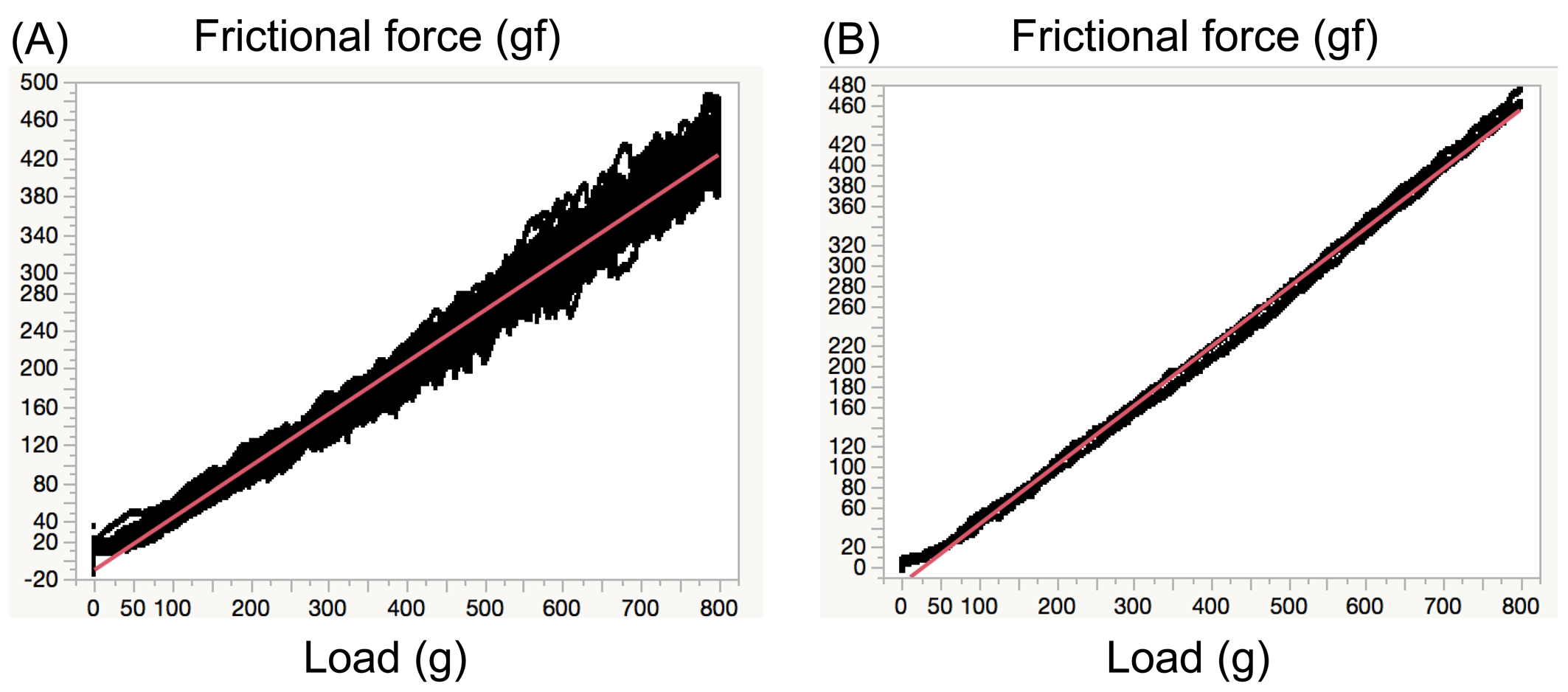

Supplement: Figure S4 — The characteristics of porcine esophageal tissue for continuous load variation friction testing were clarified, and calibration curves were prepared. The same experiments were performed on the silicone sheet. Condition; scratch over a distance of 50 mm at a speed of 0.8 mm/min while changing the normal load from 0 g to 800 g, 42 times for the porcine esophageal tissue (Figure S4-A), 3 times for the silicone sheet (Figure S4-B). The raw data were in Dataset S1 and Dataset S2. From the results, the regression line of the silicon sheet was Y = 0.588X − 16.4 (X was the load and Y was the frictional force, R2 = 0.998). The regression line of the porcine esophageal tissue was Y = 0.543X − 10.7 (X was the load and Y was the frictional force; R2 = 0.998). The regression line obtained by correcting the intercept to 0 was Y = 0.558X (R2 = 0.994) and Y = 0.523X (R2 = 0.996), and the slope ratio was 1.07. [file peerj-07-6763-s004.png]

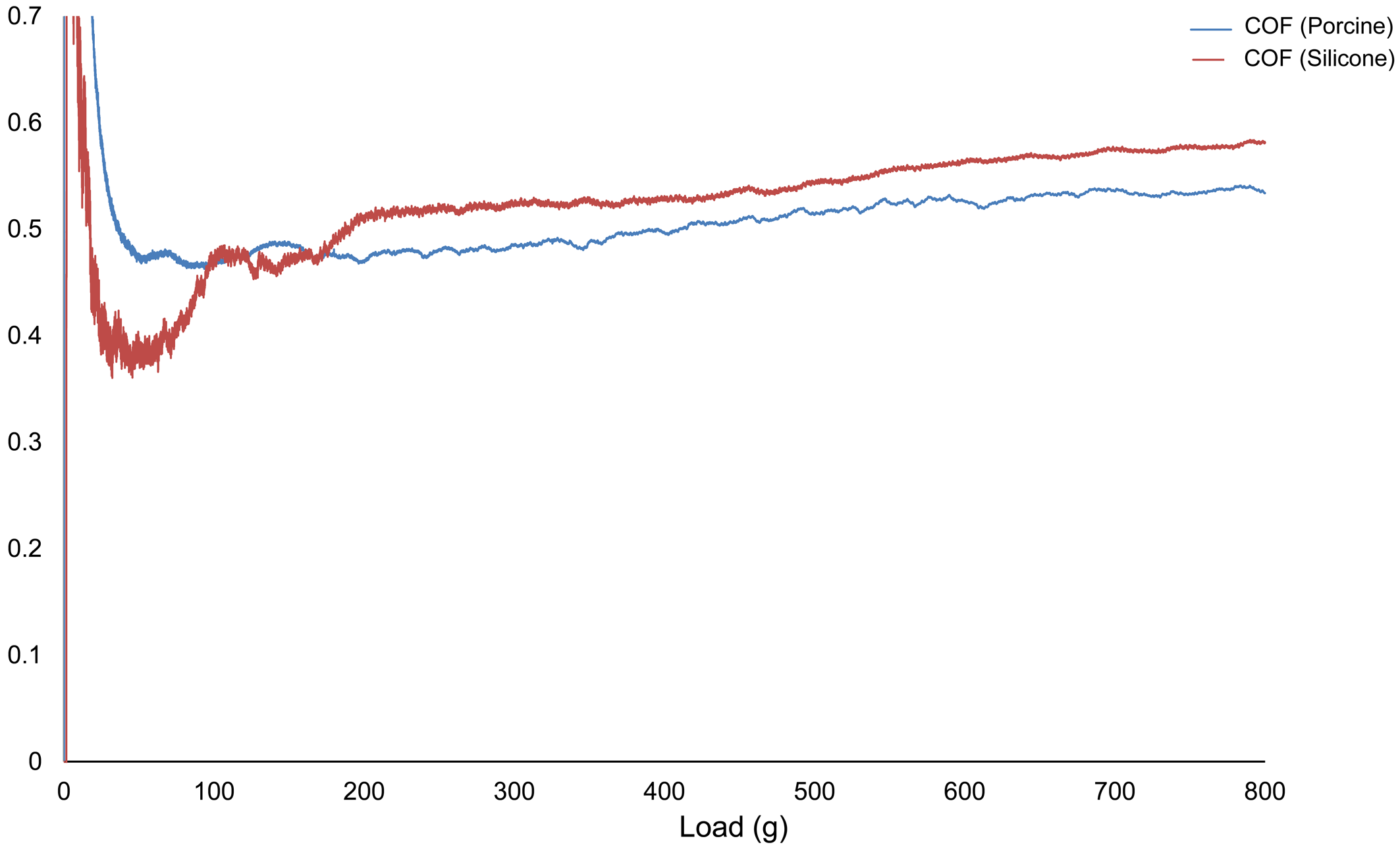

Supplement: Figure S5 — The vertical axis is COF and the horizontal axis is plotted as load in FigureS5 from FigureS4 and DatasetS3. [file peerj-07-6763-s005.png]

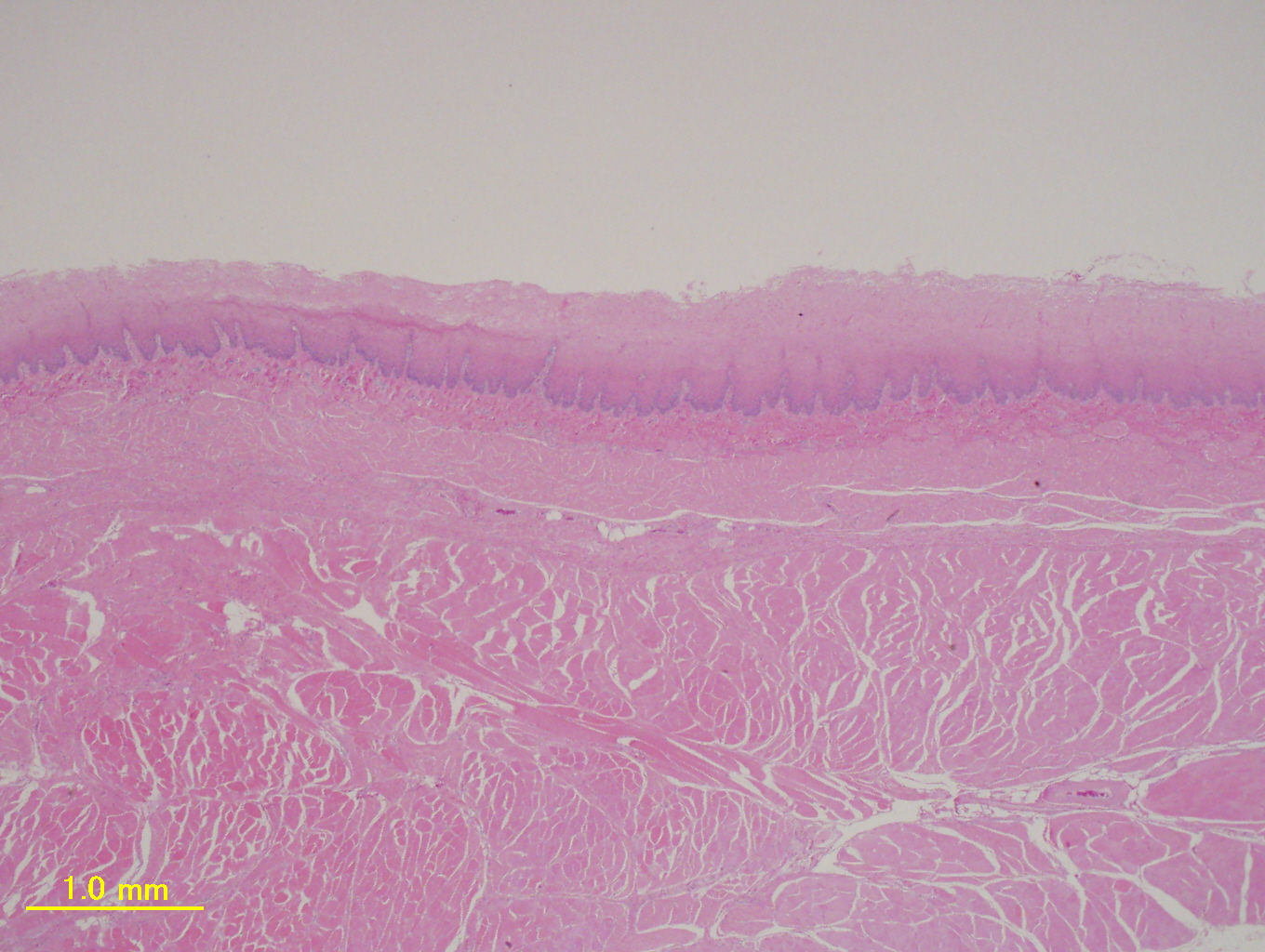

Supplement: Figure S6 — The depth of injury is stratum corneum. [file peerj-07-6763-s006.jpg]

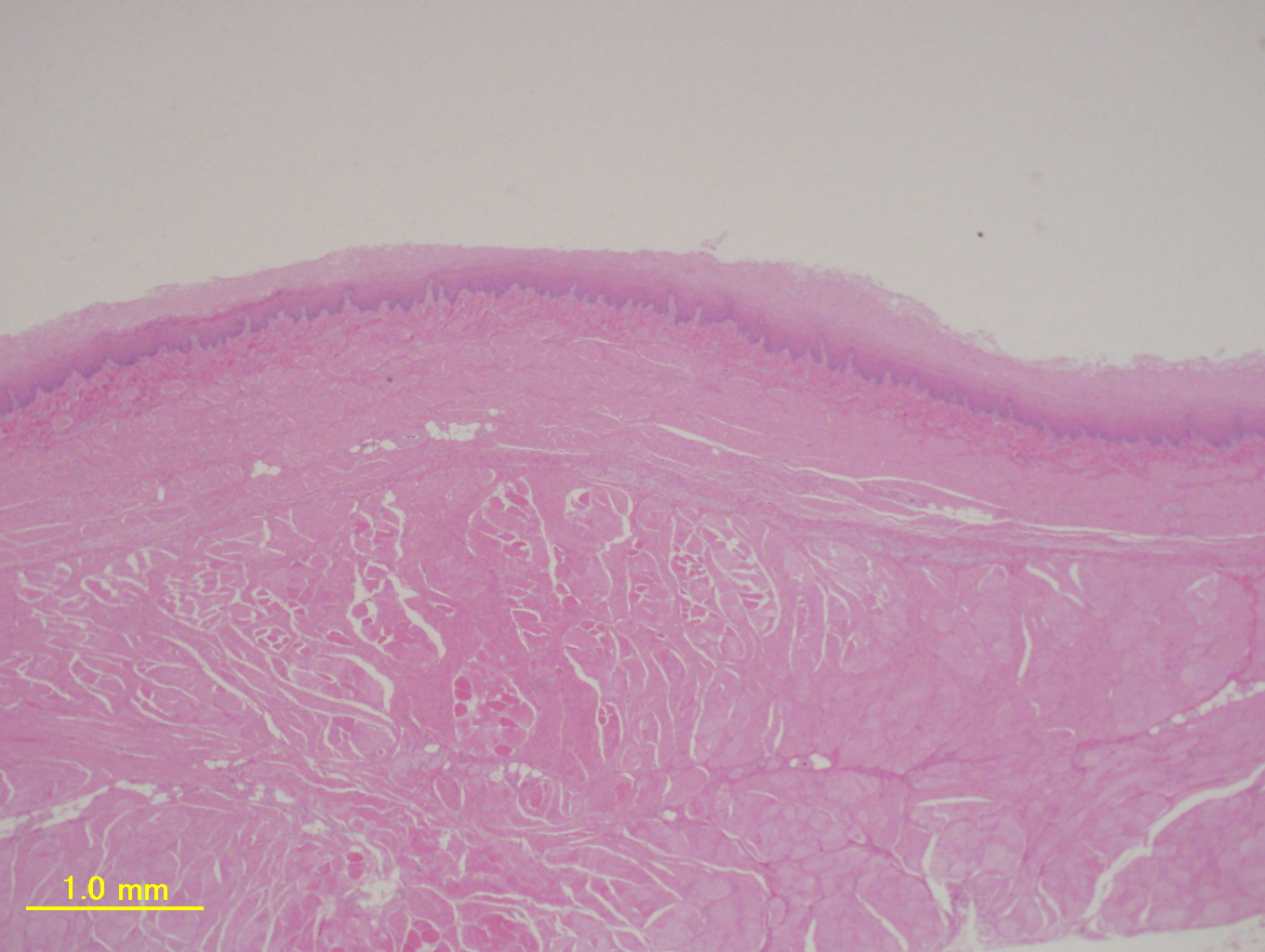

Supplement: Figure S7 — The depth of injury is basement membrane. [file peerj-07-6763-s007.jpg]

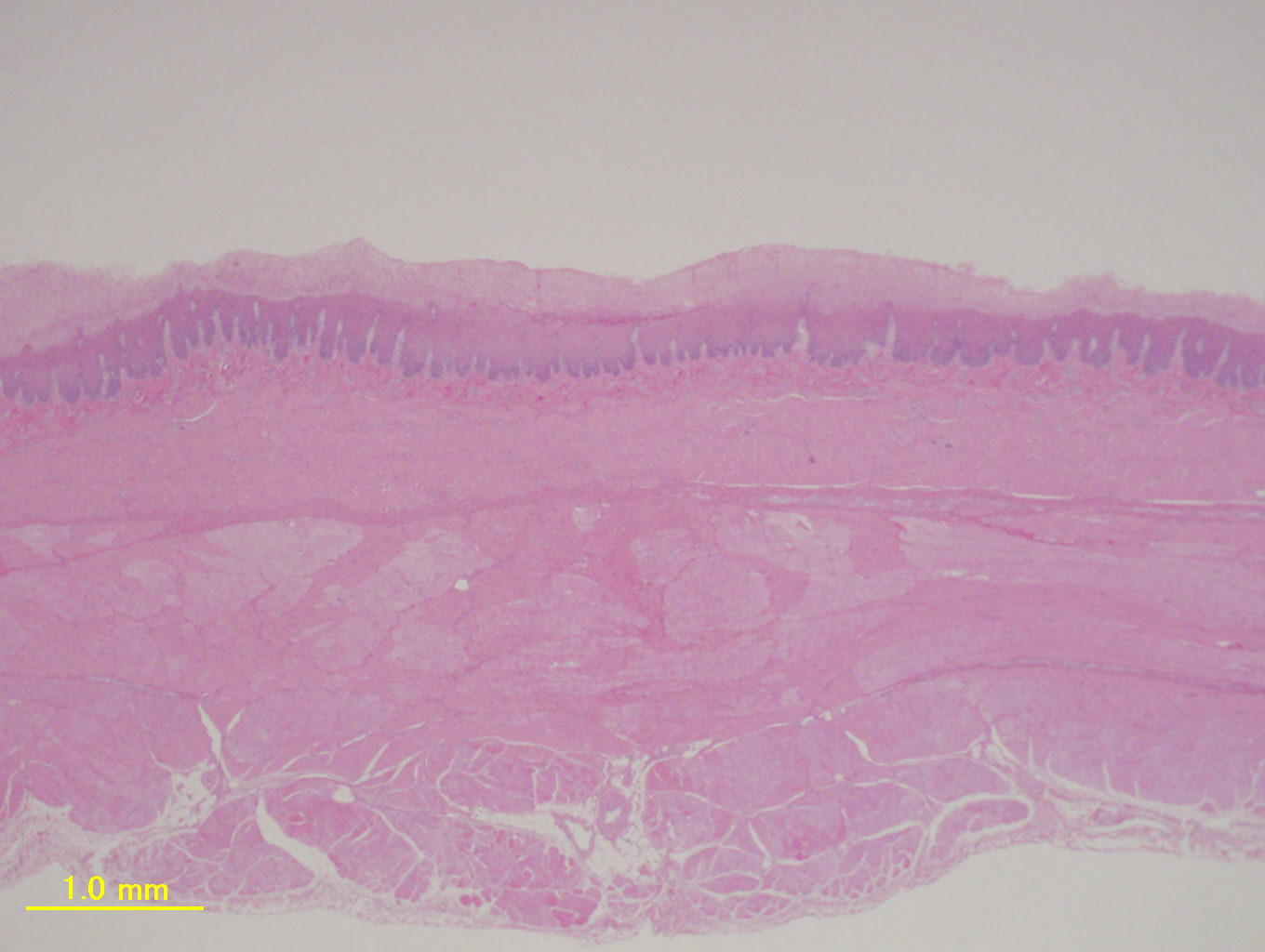

Supplement: Figure S8 — The depth of injury is stratum basale. [file peerj-07-6763-s008.jpg]

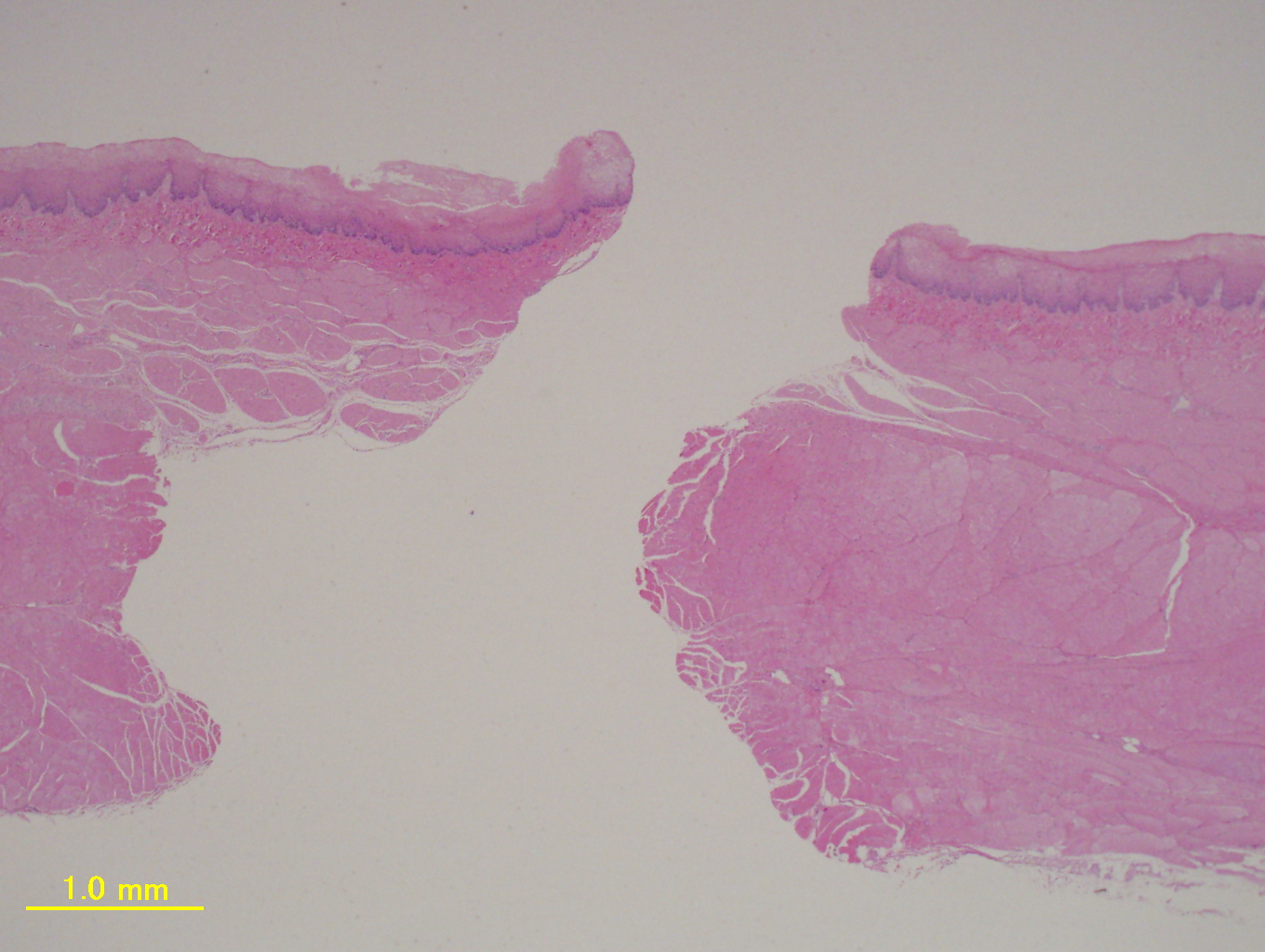

Supplement: Figure S9 — The depth of injury is stratum corneum. [file peerj-07-6763-s009.jpg]

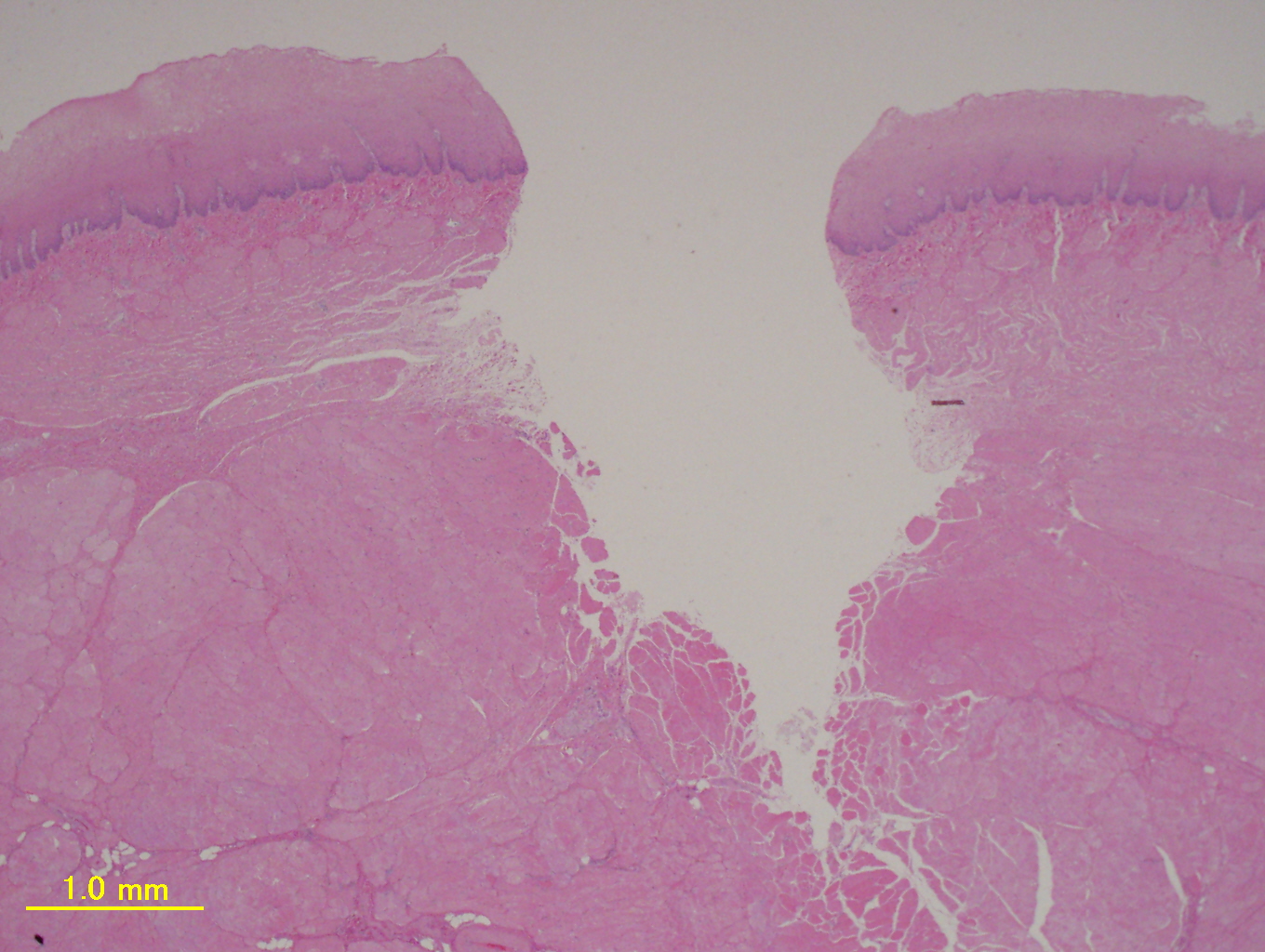

Supplement: Figure S10 — The pathological evaluation is intact. [file peerj-07-6763-s010.jpg]

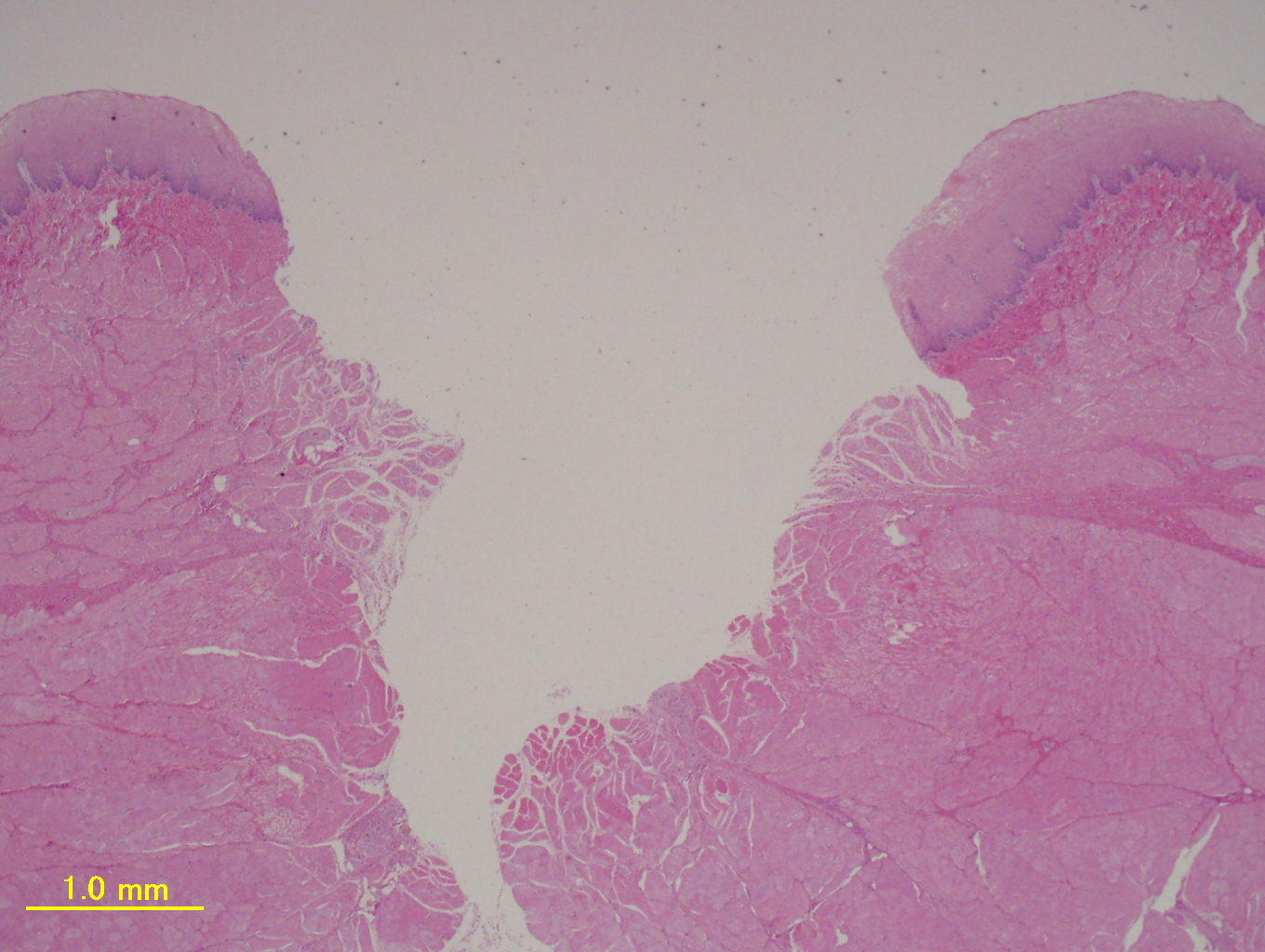

Supplement: Figure S11 — The pathological evaluation is intact. [file peerj-07-6763-s011.jpg]

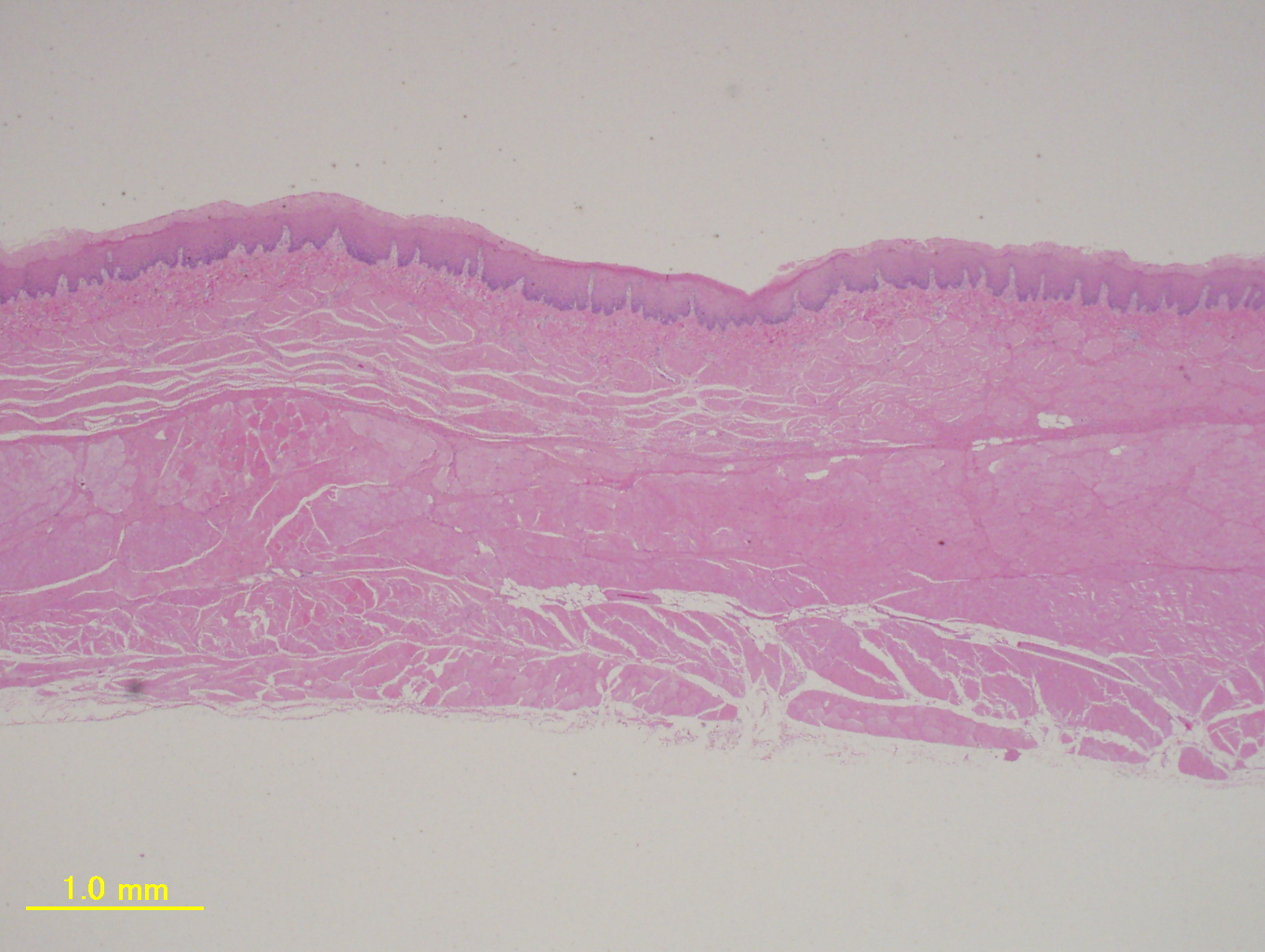

Supplement: Figure S12 — The pathological evaluation is intact. [file peerj-07-6763-s012.jpg]

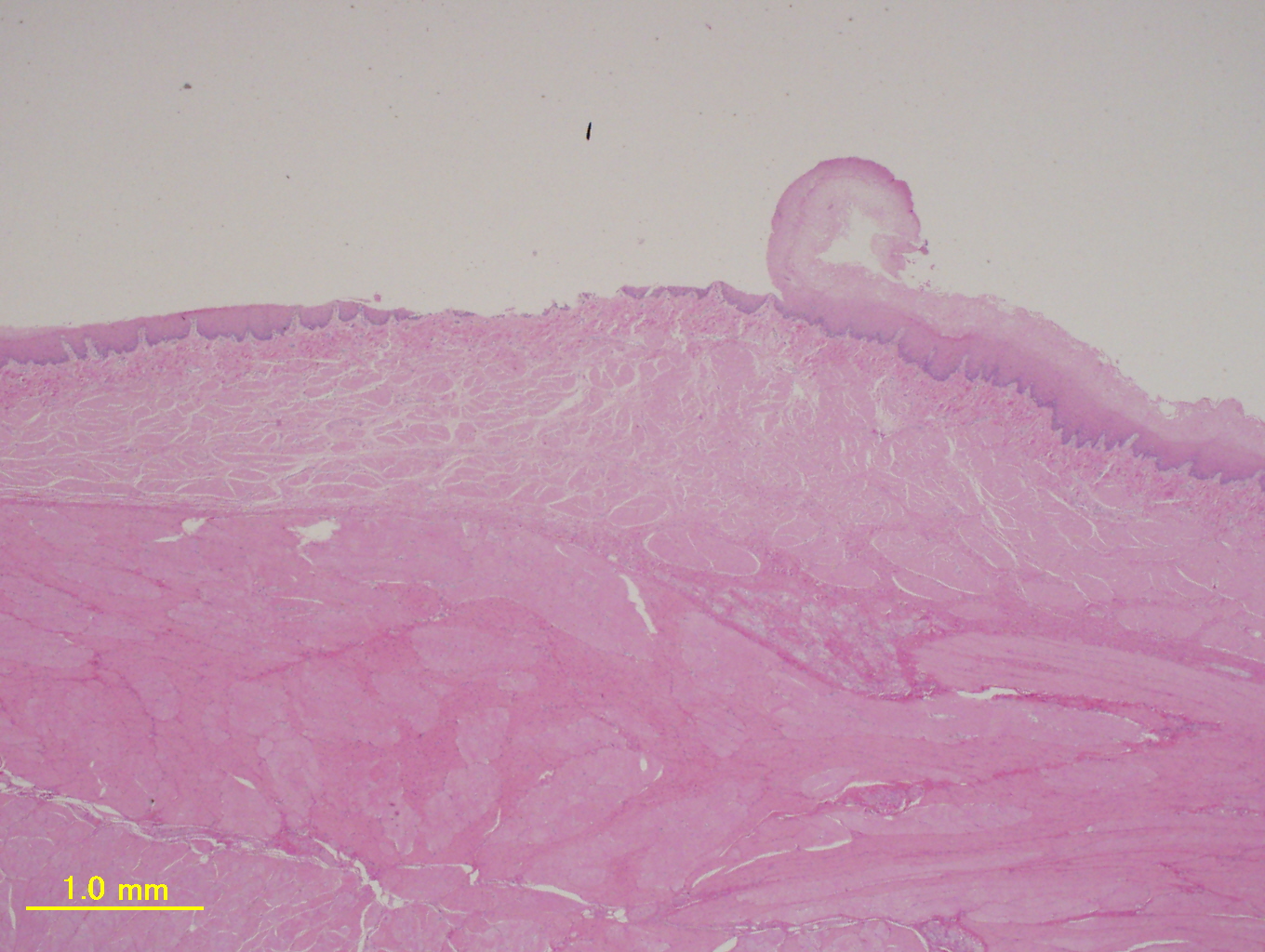

Supplement: Figure S13 — The depth of injury is stratum corneum. [file peerj-07-6763-s013.jpg]

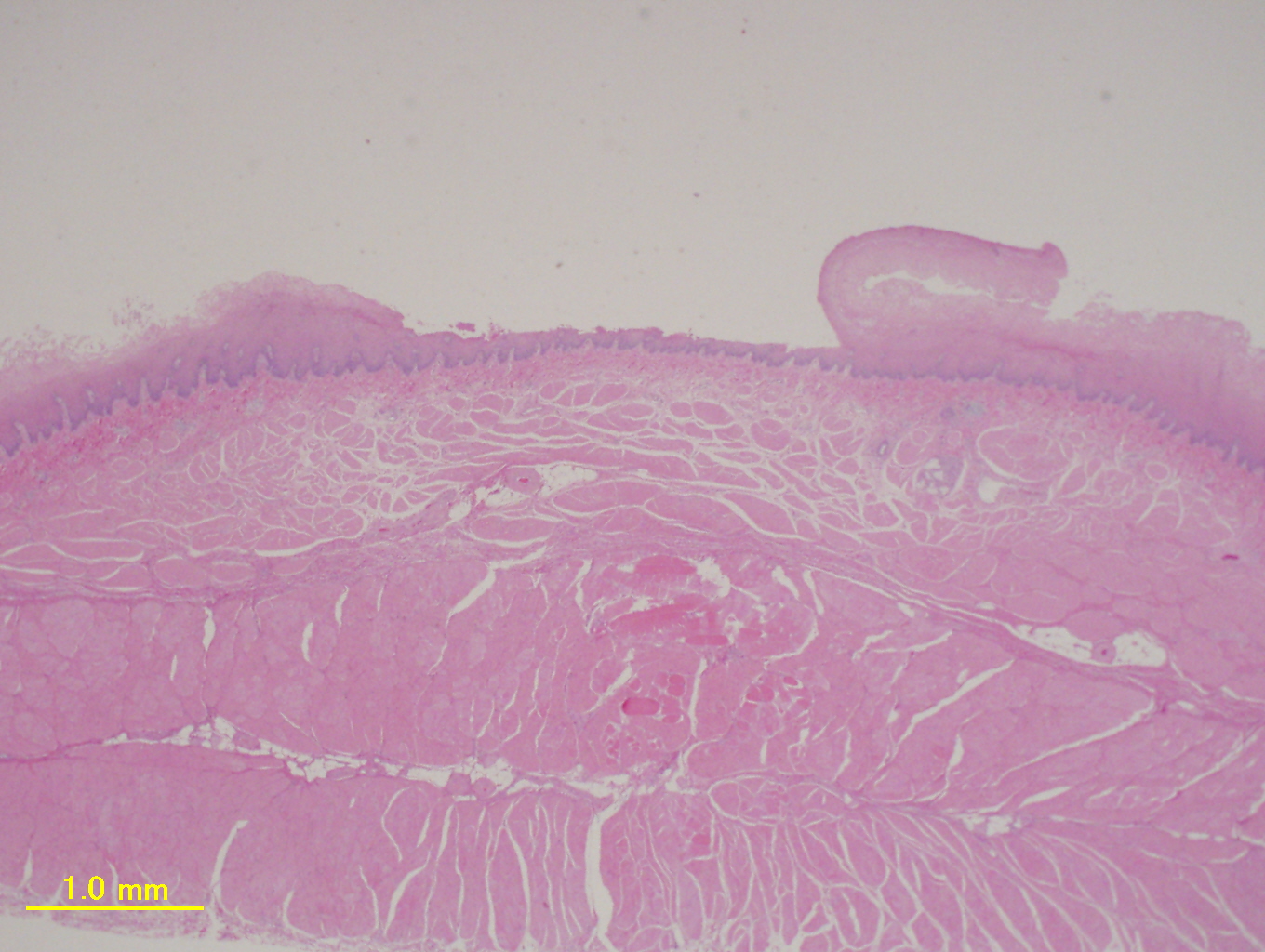

Supplement: Figure S14 — The pathological evaluation is intact. [file peerj-07-6763-s014.jpg]

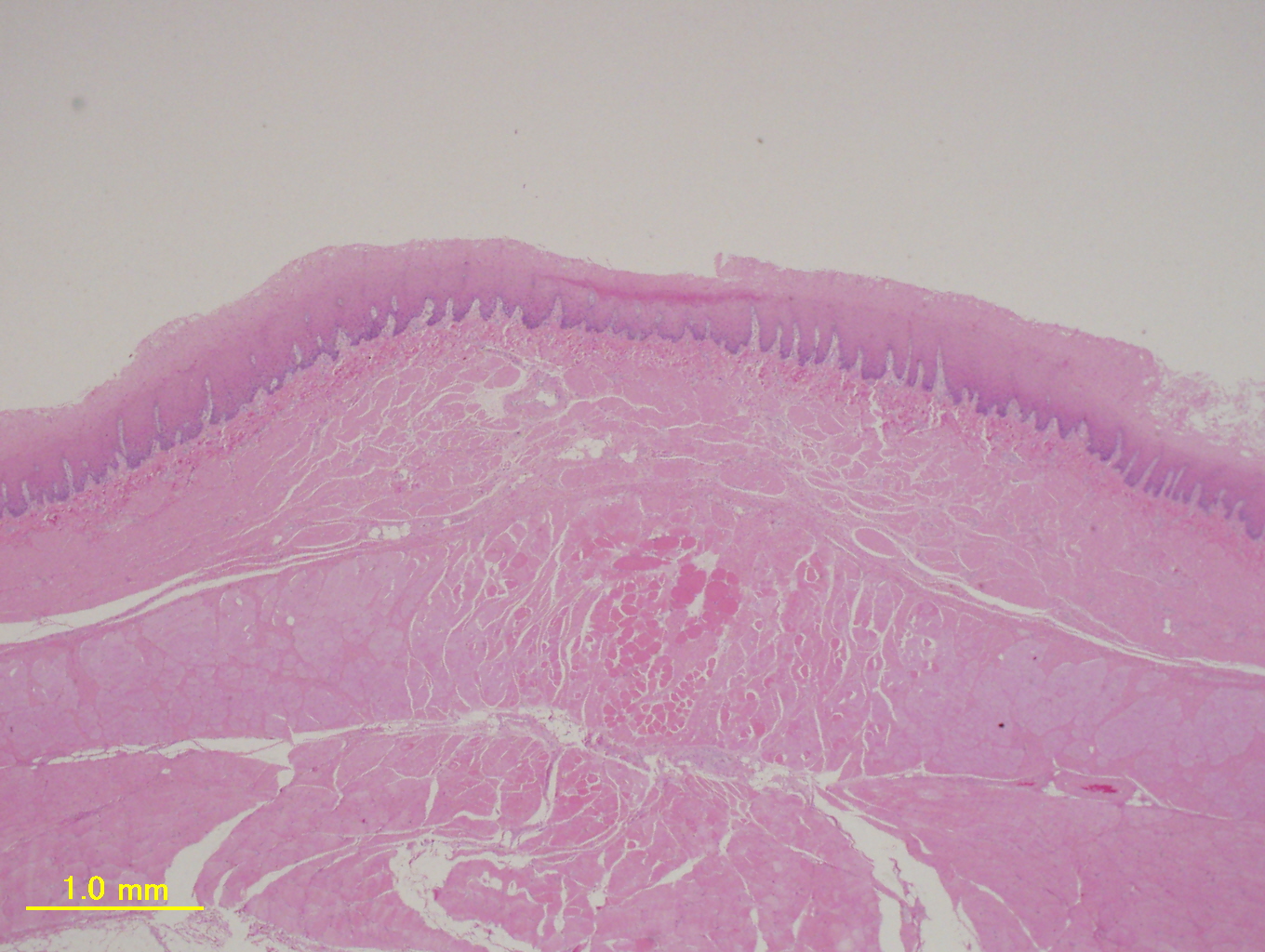

Supplement: Figure S15 — The depth of injury is adventitia. [file peerj-07-6763-s015.jpg]

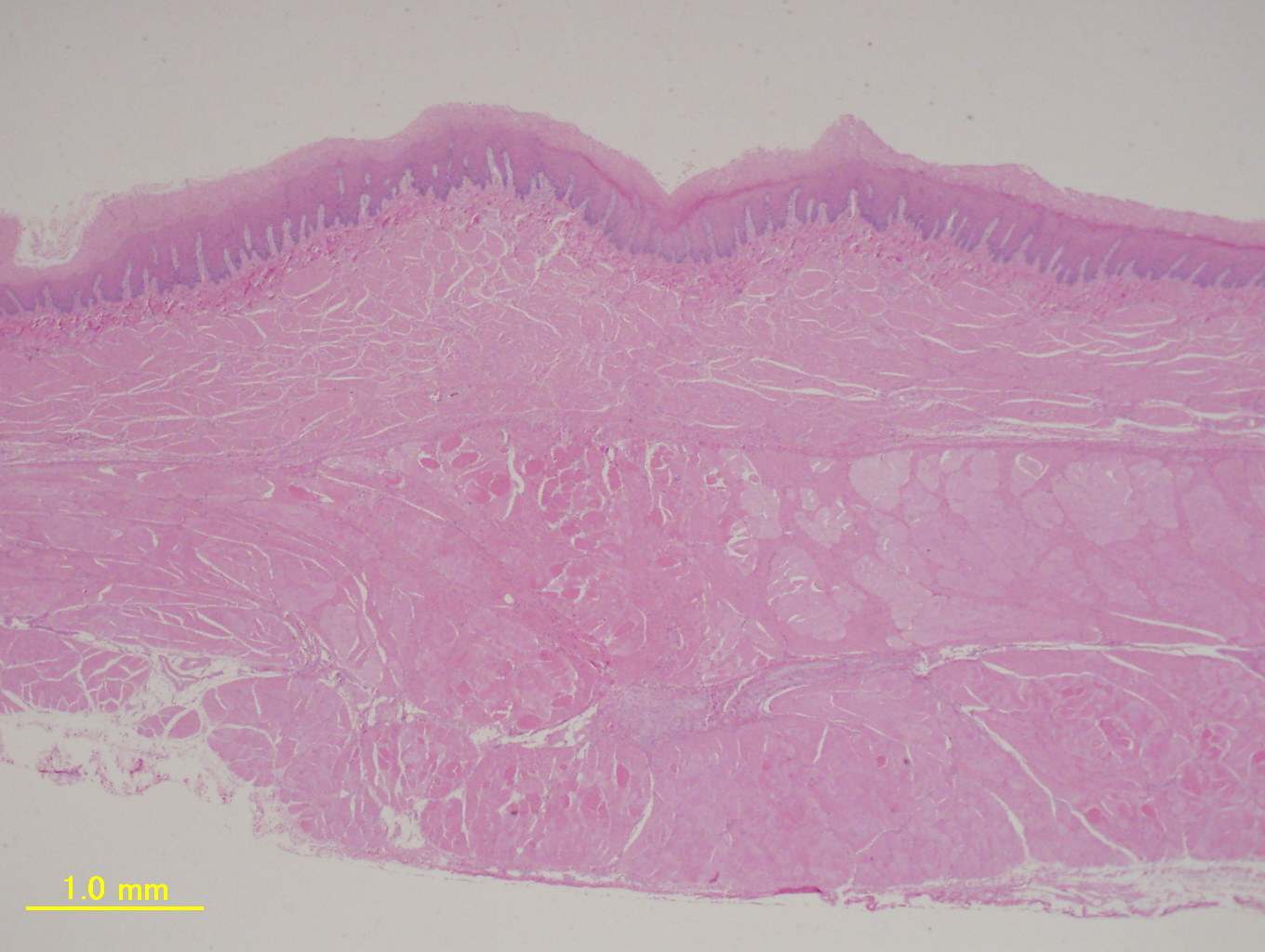

Supplement: Figure S16 — The depth of injury is adventitia. [file peerj-07-6763-s016.jpg]

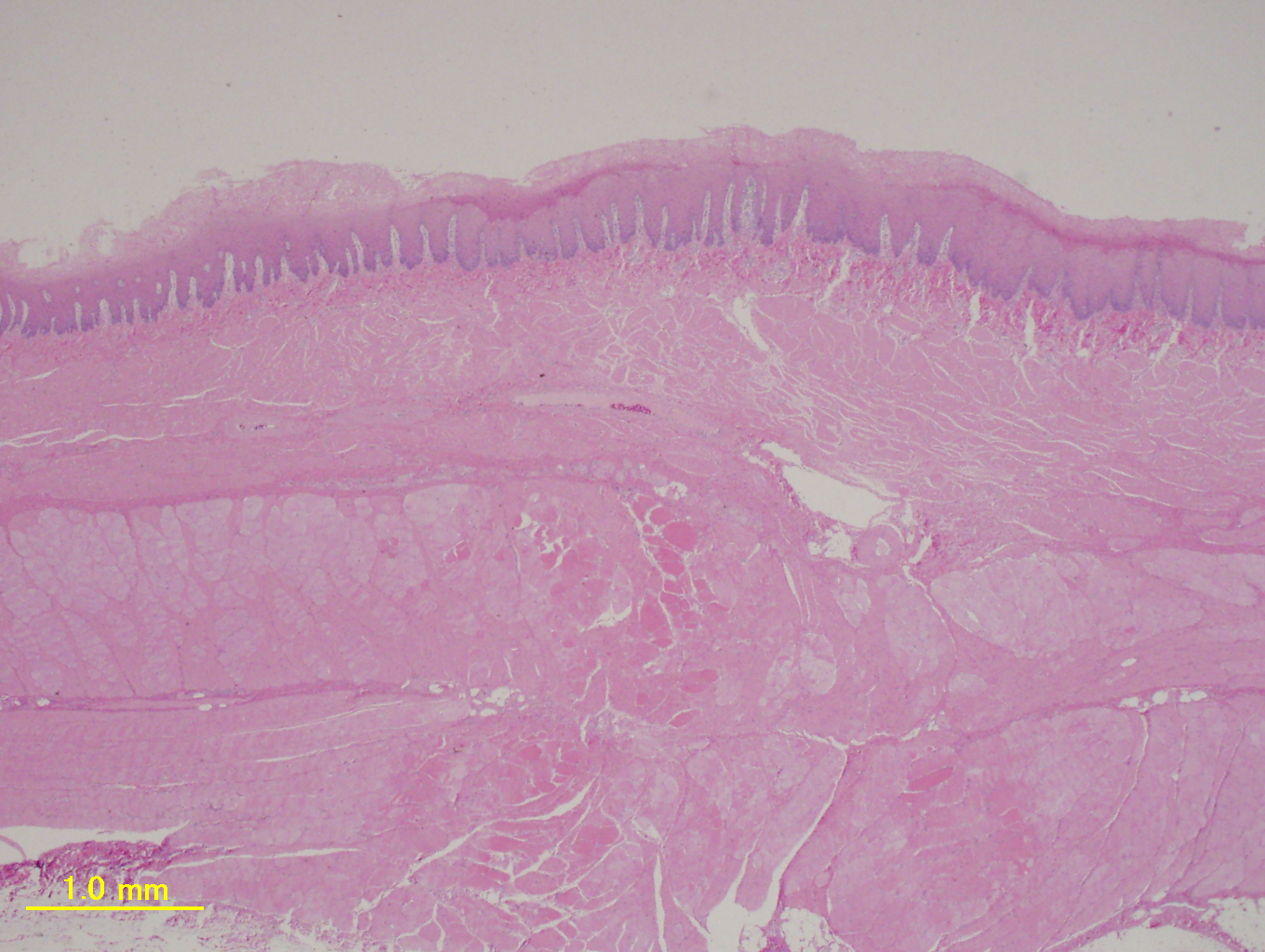

Supplement: Figure S17 — The depth of injury is adventitia. [file peerj-07-6763-s017.jpg]
